# Affordable care act: comparison of healthcare indicators among different insurance beneficiaries with new coverage eligibility

**DOI:** 10.1186/s12913-016-1362-1

**Published:** 2016-04-04

**Authors:** Young Rock Hong, Derek Holcomb, Michelyn Bhandari, Laurie Larkin

**Affiliations:** Department of Health Promotion and Administration, Eastern Kentucky University, Richmond, KY 40475 USA

**Keywords:** Affordable Care Act, Health insurance, Insurance exchanges, Medicaid, The uninsured

## Abstract

**Background:**

Health coverage in the United States will be increased to nearly universal levels under the Affordable Care Act (ACA). In order to better understand the impact of the type of health insurance and health outcomes, there is a need to examine health disparities and inequalities between the insured and the uninsured based on their eligibility for coverage.

**Methods:**

The current study used the data from the Medical Expenditure Panel Survey 2012 (MEPS). Selected health characteristics and access to care items were compared in regard to the insurance status: private, public, the uninsured, but likely eligible for Medicaid expansion (EME), and the uninsured, but likely required to purchase health plans through the health insurance exchanges (RPIE).

**Results:**

Analyses showed that 17.2 % of US adults ages 27–64 were eligible as EME and 12.9 % as RPIE in 2012. Compared to the insured groups, the uninsured who were eligible for coverage reported fewer health problems than those insured privately and publicly. However, they also reported less use of health care, including preventive health service, screenings, and unmet health care needs.

**Conclusions:**

The ACA aims to increase coverage options and access to treatment and preventive health care services for the majority of the uninsured US population. However, it may not play as significant of a role in improving health among the uninsured, in particular, those eligible for the Medicaid expansion.

## Background

With the ever-increasing percentage of Americans who are uninsured [1], as well as the rising costs of health care coverage for all Americans [2], President Barack Obama consequently signed the Patient Protection and Affordable Care Act (commonly referred to as the Affordable Care Act [ACA]) on March 23, 2010. The ACA was designed to assist both underinsured and uninsured U.S. residents by guaranteeing that all individuals have certain levels of accessibility to necessary health services [3, 4]. In addition, it was estimated that over 32 million uninsured Americans will consequently receive the minimum essential coverage under the ACA [5].

The purpose of health insurance is to facilitate sufficient access to health care, and to protect individuals as well as family members from the financial burden, especially associated with catastrophic illnesses [6, 7]. In this way, health insurance reduces the price of care faced by the health service consumers; and it will be directly connected with an increase of the demand for health care. Evidence suggests that this increase in consumption of care could result in better health status [8–10].

Considering the intriguing relationship between health insurance and health outcomes along with the increase in newly insured populations [9–11], it is imperative to examine the differences in general health status (e.g., self-reported health status, risky behaviors) and health service utilization (e.g., the number of visits to care, health screenings) between those with different types of health insurance and those who were likely to be eligible for coverage under the ACA. This would allow identification of health concerns for those with different types of insurance and to establish their health status in a broader more applicable context. Therefore, the purpose of this study was to assess health disparities and inequalities in regard to the insurance status: private, public, the uninsured but likely eligible for Medicaid expansion (EME), and the uninsured but likely required to purchase health plans through the health insurance exchanges (RPIE). A second purpose was to establish baseline information on health status and access to care prior to the ACA enactment.

## Methods

### Data

A secondary analysis was performed on data from the Household Survey Component (HC) of the Medical Expenditure Panel Survey 2012 (MEPS), a large-scale U.S. population-based survey administered by the Agency for Healthcare Research and Quality (AHRAQ). The AHRAQ conducts a year-long panel survey of over 35,000 individuals in 15,000 households, which are representative of the civilian, non-institutionalized U.S. population [12]. Consolidated MEPS data files are publically available (http://meps.ahrq.gov/mepsweb/data_stats/download_data_files.jsp).

The current study used data for adults aged 27 to 64 years who completed the self-administered questionnaire (SAQ). The elderly population, those 65 years and older, were excluded to avoid confounding with individuals using Medicare (near-universal coverage) [13, 14]. Respondents younger than 27 were also excluded to avoid effects of potential extended health insurance coverage of young adults up to the age of 26 years old; 47 % of US young adults ages 19–25 stayed or joined their parent’s health plan in 2011 [15]. These exclusion criteria resulted in a final N of 16,865 individuals with a mean age of 44.7 ± 10.74 years. Almost 54 % of the sample were women. About two-fifths of the sample reported they were Caucasian, followed by Hispanic (29.5 %), African American (20.1 %), Asian (7.9 %), and other ethnic groups (2.0 %).

The data were analyzed separately for the types of insurance status: the privately insured (*n* = 9,428), the publicly insured (*n* = 2,371), the uninsured who were likely to be required to purchase coverage through the exchanges (RPIE; *n* = 2,172), and the uninsured who were likely to be eligible for Medicaid expansion (EME; *n* = 2,894).

### Measures

Respondents were classified into four groups: (1) the first group consisted of those with private coverage purchased individually or through an employer or group. Individuals with coverage provided by the military (i.e., TRICARE and Civilian Health and Medical Program of the Department of Veterans Affairs [CHAMPVA]) were also included in this group because most of the health services under the military health program are delivered by private providers despite the special nature of the military organization [16]; (2) a public insurance group included individuals who were covered primarily through Medicaid and those with other income-determined coverage sponsored by federal or state payers. In order to identify newly insured groups with the ACA enactment, grouping criteria of the uninsured were adopted and modified for this study [17]; (3) respondents who reported no health coverage and had a family income equal to or lower than 133 % of the federal poverty level in 2012 from the US Department Health and Human Services were identified as the uninsured eligible for the Medicaid expansion (EME); (4) lastly, those who reported no health insurance and had a family income above 133 % of FPL were classified as the uninsured who would be required to purchase health insurance (RPIE).

#### Health conditions

General health indicators included self-reported physical and mental health statuses. Chronic conditions were assessed by self-reported doctor’s diagnosis (dichotomously, yes or no) comprised of high blood pressure, heart-related disease (coronary heart disease, angina, heart attack and any other heart disease), diabetes, and any type of cancer. Health related lifestyle included current smoking status and a calculated body mass index (BMI, defined as weight in kilograms divided by height squared in meters). Respondents were classified as being overweight if their calculated BMI fell between 25 and 29.99 and obese if their BMI was equal to or greater than 30 [18]. Three questions were selected that pertained to mental health, asking (a) how often the respondent felt (a) hopeless, (b) worthless, and (c) sad and had nothing to cheer him/her up. These items were recoded dichotomously as 0 (*some of the time, a little of the time, or none of the time)* and 1 (a*ll the time or most of the time).*

#### Health care utilization

Health care access included: having usual sources of care and having had a routine check-up as well as a dental check-up during the past year. Items specific to a women’s screening included: having a Pap test and breast exam within two years, and mammogram within three years. Unmet healthcare need was created to measure access to healthcare. Three questions asking the respondent’s experience when they needed health care were identified and used: unmet needs for (a) immediate care, (b) needed care/treatment/tests, and (c) making an appointment when wanted (*Cronbach’s α =* 0.701; see Table 2 notes for question details).

#### Sociodemographic factors

Eight socio-demographic variables were used to represent the basic factors related to health status and insurance: age, gender, race/ethnicity, education, marital status, employment status, family income, family size, and region (see Table 1).

**Table 1:** Demographic characteristics for the respondents by the type of coverage

	Insured		Uninsured		Significance tests			
Characteristics	Private	Public	RPIE	EME	χ^2^P			
	*n* = 9,428	*n* = 2,371	*n* = 2,172	*n* = 2,894	Private vs. Public	RPIE vs. EME	Private vs. RPIE	Public vs. EME
Age (years)	(M) 45.59 ± 0.109	(M) 45.37 ± 0.229	(M) 43.69 ± 0.199	(M) 41.41 ± 0.216	.943^a^	***^a^	***^a^	***^a^
27–45	48.8 %	48.8 %	54.5 %	66.0 %	.946	***	***	***
46–64	51.2 %	51.2 %	45.5 %	34.0 %				
Sex, Female	52.7 %	64.9 %	45.9 %	55.2 %	***	***	***	***
Race/Ethnicity					***	***	***	***
Hispanic	19.2 %	31.3 %	43.0 %	54.2 %				
White/ Non-Hispanic	51.4 %	29.6 %	30.4 %	18.6 %				
Black/ Non-Hispanic	17.6 %	31.4 %	17.3 %	22.7 %				
Asian	9.7 %	5.1 %	7.4 %	3.7 %				
Others	2.1 %	2.6 %	1.9 %	0.8 %				
Education, College or Higher Education (more than 12 years)	67.0 %	29.1 %	40.5 %	26.5 %	***	***	***	.05
Married	68.4 %	33.9 %	53.6 %	42.2 %	***	***	***	***
Employed	85.1 %	28.6 %	73.5 %	52.0 %	***	***	***	***
Family Income					***	***	***	***
Low income (<200 % FPL)	17.5 %	82.0 %	33.8 %	100 %				
Middle income (≥ 200 to < 400 % FPL)	34.5 %	14.2 %	47.9 %	.				
High income (≥ 400 % FPL)	48.0 %	3.8 %	18.3 %	.				
Family Size					***	***	***	***
< 3	41.1 %	42.6 %	38.7 %	31.4 %				
3 to 4	42.4 %	36.0 %	37.8 %	33.9 %				
5 to 7	15.8 %	19.4 %	21.2 %	30.7 %				
> 7	0.7 %	2.0 %	2.3 %	4.0 %				
Region					***	***	***	***
Northeast	16 %	26.7 %	12.6 %	10.9 %				
Midwest	21.2 %	17.0 %	14.3 %	12.4 %				
South	35.6 %	31.2 %	42.0 %	49.8 %				
West	27.3 %	25.2 %	31.1 %	26.9 %				

*Note.* * *p* < .05, ** *p* < .01, *** *p* < .001, based on *χ* ^2^ analysis; Data from Medical Expenditure Panel Survey (MEPS) 2012; Numbers are unweighted and percentages do not always equal 100 due to rounding or missing data

*RPIE* the Uninsured Who Will Likely Be Required to Purchase Health Insurance through the Exchanges under the ACA Enactment, *EME* the Uninsured Who Will Likely Be Eligible for Medicaid Expansion, *FPL* Federal Poverty Level in 2012

^a^ Tests for differences between insurance groups based on the analysis of variance (ANOVA)

### Statistical analyses

The Statistical Package for the Social Sciences version 22 (IBM Corp., Armonk, NY, USA) was used to analyze the data in 2014. Descriptive statistics were calculated to summarize the proportions of categorical variables and tests of statistical significance were performed using the chi-square test or Fisher’s exact test in order to compare differences in socio-demographics, self-reported health status, leading health indicators, and access to care between the groups. Multivariate logistic regression was performed to assess the association with primary health status indicators, prevalence of chronic conditions and cancer, and access to care between the groups. Adjusted odds ratios were generated by controlling for age, gender, race/ethnicity, education, marital status, employment status, family income, family size, and region.

## Results

### Insurance-type difference in socio-demographics

Table 1 presents information on the distribution of socio-demographic characteristics for the four insurance types. All variables were associated with insurance status (based on Chi-square analysis, *p* < 0.001). The majority of respondents (70 %) had health insurance coverage. Over half of respondents (56 %) had employment-based coverage, and about 14.1 % reported they were covered by publicly funded insurance. Thirty percent reported they had no insurance coverage. Of the sample who were uninsured and provided data on family income and the number of family members to determine eligibility for the Medicaid expansion (*n* = 5,066), 57.1 % were likely to be eligible for the Medicaid expansion (EME; accounting for 17.2 % of the total sample) and 42.9 % were likely to be required to purchase coverage through health insurance exchanges (RPIE; 12.8 % of the total). Table 1 shows more detail on socio-demographic characteristics by the type of insurance and eligibility for coverage.

### Insurance-type difference in health characteristics

Table 2 shows the bivariate results comparing the type of insurance with health status attributes and health service utilization. Table 3 displays the multiple logistic regression results with the odds of having selected health conditions and experiencing health services by the type of insurance coverage (Private vs. Public, RPIE vs. EME, Private vs. RPIE, and Public vs. EME). Adjusted odds ratios (AORs) controlling for important socio-demographics (age, gender, race/ethnicity, education, marital status, employment status, family income, family size, and region) and their 95 % confidence intervals (CI) are presented.

**Table 2:** Bivariate analysis on health characteristics and health service use

	Insured		Uninsured		Significance tests			
Characteristics	Private	Public	RPIE	EME	χ^*2*^P			
	*n* = 9,428	*n* = 2,371	*n* = 2,172	*n* = 2,894	Private vs. Public	RPIE vs. EME	Private vs. RPIE	Public vs. EME
General Health								
Fair/Poor, self-reported health	9.7 %	39.6 %	16.7 %	23.9 %	***	***	***	***
Chronic Conditions								
Heart-related Disease	8.9 %	17.7 %	6.6 %	6.8 %	***	.851	***	***
Diabetes	7.5 %	18.1 %	6.5 %	7.9 %	***	.051	.078	***
Cancer	6.7 %	7.3 %	3.6 %	3.0 %	.332	.238	***	***
Health-Related Lifestyle								
Overweight^a^	35.0 %	30.8 %	36.6 %	34.4 %	***	.107	.103	*
Obese^b^	32.3 %	43.0 %	31.4 %	34.5 %	***	*	.390	***
Smoking	14.7 %	29.8 %	21.6 %	23.8 %	***	.064	***	***
Mental Health^c^								
Felt Hopeless all the time or most of the time	2.3 %	12.7 %	4.1 %	7.1 %	***	***	***	***
Felt Sad all the time or most of the time	1.6 %	10.4 %	3.1 %	5.8 %	***	***	***	***
Felt Worthless all the time or most of the time	2.1 %	10.5 %	3.6 %	5.3 %	***	**	***	***
Health Care Access								
Have Usual Source of Care	79.0 %	80.4 %	55.1 %	57.8 %	.135	.054	***	***
Routine Check-up, a prior year	67.5 %	74.9 %	39.1 %	37.3 %	***	.179	***	***
Dental Check-up, a prior year	71.0 %	42.3 %	39.9 %	31.4 %	***	***	***	***
Visits to Medical Offices^d^					***	*	***	***
0	29.0 %	22.4 %	59.3 %	62.6 %				
1	19.2 %	12.9 %	13.7 %	11.8 %				
2	16.0 %	13.3 %	8.9 %	9.1 %				
3	11.3 %	11.2 %	6.2 %	5.3 %				
4	8.2 %	11.6 %	4.7 %	3.4 %				
5 to 9	10.7 %	15.5 %	5.0 %	4.6 %				
≥ 10	5.6 %	13.1 %	2.2 %	3.2 %				
Women’s Screening								
Pap test, prior 3 years^e^	89.1 %	85.0 %	76.3 %	76.2 %	***	.963	***	***
Breast Exam, prior 2 years^f^	85.4 %	78.6 %	65.7 %	61.5 %	***	*	***	***
Mammogram, prior 2 years^g^	62.2 %	53.6 %	40.4 %	33.6 %	***	**	***	***
Unmet Health Needs								
Immediate Care/Treatment Due to Injuries or Illnesses^h^	13.7 %	19.5 %	28.9 %	35.9 %	***	*	***	***
Needed Care/Treatment/Tests^i^	7.1 %	15.7 %	22.3 %	30.9 %	***	**	***	***
Appointment When Wanted^j^	15.5 %	18.4 %	26.5 %	31.6 %	**	*	***	***

*Note.* * *p* < .05, ** *p* < .01, *** *p* < .001, based on *χ* ^2^ analysis; Data from Medical Expenditure Panel Survey (MEPS) 2012; Numbers are unweighted and percentages do not always equal 100 due to rounding or missing data

*RPIE* the Uninsured Who Will Likely Be Required to Purchase Health Insurance through the Exchanges under the ACA Enactment, *EME* the Uninsured Who Will Likely Be Eligible for Medicaid Expansion

^a^ Defined as a BMI ≥ 25 to < 30

^b^ Defined as a BMI ≥ 30

^c^ The percentage of individuals who reported they felt most of the time during the past 30 days

^d^ The number of visits to doctor’s office or clinic in the past 12 months, emergency visits are not include

^e^ Analysis was restricted to women aged 27–64 years (Pap test; *n* = 8,588, Breast exam; *n* = 8,618); numbers are not same due to missing data

^f^ Analysis was restricted to women aged 29–64 years (*n* = 7917)

^g^ The percentage of individuals who answered never or sometimes to the following question: “In the last 12 months, when you needed care right away how often did you get care as soon as you though you needed?”

^h^ The percentage of individuals who answered never or sometimes to the following questions: “In the last 12 months, how often was it easy to get the care, tests, or treatment you or a doctor believed necessary?”

^i^ The percentage of individuals who answered never or sometimes to the following questions: “In the last 12 months, not counting the times you needed care right away, how often did you get an appointment for your health care at a doctor’s office or clinic as soon as you thought you needed?”

**Table 3:** Multivariate analysis on health characteristics and health service use

	Private vs. Public		RPIE vs. EME		Private vs. RPIE		Public vs. EME	
Characteristics	Odds ratio (95 % CI)	Adjusted odds ratio (95 % CI)	Odds ratio (95 % CI)	Adjusted odds ratio (95 % CI)	Odds ratio (95 % CI)	Adjusted odds ratio (95 % CI)	Odds ratio (95 % CI)	Adjusted odds ratio (95 % CI)
General Health								
Fair/Poor, self-reported health	0.164*** (0.147, 0.183)	0.469*** (0.400, 0.550)	0.639*** (0.556, 0.735)	0.856 (0.699, 1.048)	0.537*** (0.476, 0.605)	0.823** (0.719, 0.942)	2.091*** (1.836, 2.380)	2.020*** (1.727, 2.363)
Chronic Conditions								
Heart-related Disease	0.453*** (0.399, 0.515)	0.693*** (0.566, 0.849)	0.979 (0.784, 1.223)	0.715 (0.507, 1.009)	1.369*** (1.164, 1.611)	1.304** (1.089, 1.561)	2.957*** (2.426, 3.604)	2.221*** (1.767, 2.792)
Diabetes	0.366*** (0.322, 0.417)	0.548*** (0.448–0.671)	0.808 (0.652, 1.001)	1.019 (0.751, 1.382)	1.162 (0.983, 1.372)	1.464*** (1.216, 1.761)	2.561*** (2.123, 3.090)	2.439*** (1.951, 3.050)
Cancer	0.917 (0.770, 1.092)	1.060 (0.812, 1.384)	1.208 (0.882, 1.656)	0.913 (0.569, 1.466)	1.924*** (1.557, 2.378)	1.412** (1.121, 1.777)	2.535*** (1.894, 3.394)	1.773** (1.270, 2.475)
Health-Related Lifestyle								
Overweight	1.206*** (1.095, 1.329)	0.957 (0.830, 1.103)	1.100 (0.979, 1.236)	1.002 (0.846, 1.186)	0.930 (0.853, 1.015)	0.959 (0.870, 1.057)	0.849* (0.749, 0.961)	0.999 (0.860, 1.160)
Obese	0.633*** (0.577, 0.694)	0.858* (0.748, 0.984)	0.871* (0.774, 0.981)	1.094 (.924, 1.296)	1.040 (0.951, 1.138)	1.209*** (1.094, 1.337)	1.432*** (1.270, 1.615)	1.366*** (1.183, 1.576)
Smoking	0.404*** (0.364, 0.449)	0.705*** (0.600, 0.828)	0.882 (0.773, 1.007)	0.797* (0.646, 0.984)	0.625*** (0.562, 0.694)	0.727*** (0.644, 0.820)	1.364*** (1.195, 1.556)	1.112 (0.944, 1.310)
Mental Health								
Felt Hopeless all the time or most of the time	0.163*** (0.136, 0,195)	0.515*** (0.396, 0.669)	0.558*** (0.436, 0.713)	0.698 (0.486, 1.005)	0.555*** (0.442, 0.696)	0.799 (0.623, 1.026)	1.899*** (1.550, 2.327)	1.568*** (1.245, 1.975)
Felt Sad all the time or most of the time	0.142*** (0.115, 0.174)	0.539*** (0.400–0.727)	0.523*** (0.397, 0.688)	0.692 (0.463, 1.035)	0.508*** (0.391, 0.661)	0.721* (0.540, 0.963)	1.873*** (1.499, 2.339)	1.491** (1.158, 1.921)
Felt Worthless all the time or most of the time	0.186*** (0.153, 0.225)	0.286*** (0.221, 0.369)	0.654** (0.499, 0.858)	0.721 (0.475, 1.094)	0.587*** (0.461, 0.748)	0.774 (0.594, 1.009)	2.070*** (1.647, 2.602)	1.557** (1.202, 2.018)
Health Care Access								
Have Usual Source of Care	0.918 (0.820, 1.027)	0.835* (0.708, 0.985)	1.117 (0.998, 1.249)	1.039 (0.880, 1.227)	4.631*** (4.239, 5.059)	3.841*** (3.473, 4.247)	5.635*** (4.936, 6.434)	4.473*** (3.829, 5.227)
Routine Check-up, a prior year	0.697 (0.629, 0.772)	0.720*** (0.620, 0.835)	1.082 (0.965, 1.213)	1.081 (0.914, 1.279)	3.235*** (2.967, 3.526)	2.908*** (2.638, 3.205)	5.019*** (4.419, 5.700)	3.929*** (3.383, 4.563)
Dental Check-up, a prior year	3.337*** (3.041, 3.662)	1.993*** (1.738, 2.286)	1.451*** (1.291, 1.632)	1.419*** (1.198, 1.681)	3.690*** (3.384, 4.024)	2.670*** (2.424, 2.941)	1.605*** (1.421, 1.813)	1.547*** (1.334, 1.795)
Women’s Screening								
Pap test, prior 3 years^b^	1.431*** (1.208, 1.696)	1.094 (0.849, 1.411)	1.005 (0.831, 1.214)	1.102 (0.834, 1.456)	2.523*** (2.150, 2.961)	2.436*** (2.036, 2.914)	1.771*** (1.453, 2.157)	1.889*** (1.490, 2.393)
Breast Exam, prior 2 years^b^	1.594*** (1.375, 1.849)	1.047 (0.841, 1.305)	1.200* (1.015, 1.418)	1.186 (0.933, 1.508)	3.059*** (2.653, 3.529)	2.746*** (2.348, 3.210)	2.303*** (1.939, 2.734)	2.045*** (1.674, 2.498)
Mammogram, prior 2 years^c^	1.424*** (1.258, 1.611)	1.066 (0.868, 1.309)	1.341** (1.124, 1.600)	1.089 (0.837, 1.416)	2.426*** (2.122, 2.773)	2.665*** (2.269, 3.130)	2.286*** (1.931, 2.705)	1.848*** (1.500, 2.277)
Unmet Health Needs								
Immediate Care/Treatment^d^	0.655*** (0.537, 0.798)	0.711* (0.521, 0.969)	0.723* (0.556, 0.942)	0.849 (0.580, 1.242)	0.390*** (0.314, 0.484)	0.466*** (0.367, 0.592)	0.430*** (0.336, 0.552)	0.440*** (0.332, 0.583)
Needed Care/Treatment/Tests^e^	0.411*** (0.341, 0.495)	0.622** (0.470, 0.824)	0.639** (0.495, 0.826)	0.830 (0.576, 1.194)	0.268*** (0.219, 0.328)	0.341*** (0.273, 0.424)	0.416*** (0.326, 0.531)	0.408*** (0.309, 0.538)
Appointment When Wanted^f^	0.818** (0.707, 0.947)	0.980 (0.787, 1.222)	0.782* (0.629, 0.971)	0.741 (0.539, 1.019)	0.510*** (0.435, 0.598)	0.593*** (0.498, 0.705)	0.487*** (0.396, 0.600)	0.540*** (0.427, 0.685)

*Note.* * *p* < .05, ** *p* < .01, *** *p* < .001; Data from Medical Expenditure Panel Survey (MEPS) 2012; Adjusted odds ratios were obtained from the multiple logistic regression controlling for age, gender, race/ethnicity, family income, education, marital status, region and family size

Analyses revealed the publicly insured were significantly more likely than the privately insured or other two uninsured groups to report poor health (see Table 2 for proportions of health indicators). Specifically, the adjusted odds ratio (AOR) for the privately insured were 0.47 times (95 % confidence interval [CI] = 0.40–0.55) less likely than those publicly covered and were 0.82 times (CI = 0.72–0.94) less likely than the uninsured who were require to purchase coverage through the insurance exchanges (RPIE) to report fair/poor health status (see Table 3 for odds ratios). However, a comparison of both uninsured groups (RPIE vs. EME) showed their difference on general health was not statistically significant after important individual characteristics were controlled for. Comparing the publicly insured with the uninsured who were EME (i.e., prospective publicly insured), the publicly insured were twice as likely to report poor health as the EME were (AOR = 2.02; CI = 1.73–2.36).

For chronic conditions, the insured groups were more likely to have a health-related disease and diabetes than the uninsured groups with the exception of cancer. The odds of the privately insured reporting heart disease were 1.30 times (CI = 1.1–1.56) higher than those who were RPIE. The publicly insured were 2.43 times (CI = 1.95–3.05) more likely than those who were EME to have diabetes. For health-related lifestyle, we found that the insured groups had a higher likelihood of being obese than the uninsured groups. Comparing both insured groups, the privately insured were less likely to report being obese (AOR = 0.86; CI = 0.75–0.98) or being a smoker (AOR = 0.71; CI = 0.60–0.83) than the publicly insured. Finally, regarding mental health, those insured publicly were significantly more likely to report feeling hopeless, sad, and worthless when compared to the other three groups (see Table 2).

### Insurance-type difference in access to care and health service utilization

With regard to access to care, the insured were more likely to have a usual source of care (USC) than were the uninsured. Among those insured, the publicly insured were 1.39 times (inverted; CI = 1.61–1.19) more likely than the privately insured to have a routine check-up during the past year. The difference in routine check-ups between the RPIE and EME was not statistically significant. Compared with the insured groups, the uninsured groups found it harder to get a dental check-up during the past year. The privately insured were 2.67 times more likely than the uninsured who were RPIE to have a dental check-up; and the publicly insured were 1.55 times more likely than the uninsured who were EME to have a dental check-up during the past year.

In terms of the number of visit to medical offices, more than half of both the RPIE (59.3 %) and EME (62.6 %) had no visit during the past 12 months, compared with 29 % of the privately insured and 22.4 % of the publicly insured (see Table 2).

For women’s cancer screening, insured women were more likely to report having had all three screenings than were uninsured women. Women who were privately insured were more than twice as likely to have had a Pap test, breast exam, and mammogram as uninsured women who were likely to have coverage through insurance exchanges. Whether the type of insurance was private or public did not significantly affect women’s health screenings after controlling for the important individual characteristics (see Table 3). Regarding unmet health care needs, the uninsured groups were more likely to find it difficult to receive immediate care and needed health services. For instance, the adjusted odds of the uninsured who were RPIE reporting difficulties to obtain needed health services were 2.94 times (inverted; CI = 2.38–3.70) higher than the privately insured. The uninsured who were EME were 2.44 times (inverted; CI = 1.85–3.23) more likely to find it harder to get needed health services than were the publicly insured. The type of insurance (whether private or public) was found to affect experiencing unmet health service needs with the exception of making appointments.

## Discussion

This study provided an opportunity to examine the socio-demographic and health characteristics, health service utilization, and needs for health care services among those who reported having different types of health insurance coverage and those who were likely to be eligible for coverage under the Affordable Care Act (ACA). The findings shed light on the gap between those insured and eligibility groups, and contribute to a better understanding of the health needs of the uninsured with eligibility for new coverage. This may foster the identification of future solutions, including special interventions for individuals who have different insurance status; changes in current enactments at the state and federal level; and a reduction of identified barriers for those newly eligible for coverage.

We found that the Affordable Care Act is likely to have a substantial impact on uninsured U.S. adults. Based on the findings, 77.7 % of those who were uninsured would be likely to have significant subsidies and would be more likely to be covered under the ACA. This would represent 37.3 million Americans based on a current estimated uninsured rate of 7.6 %. Recent studies show that a total of 25.5 million Americans have gained coverage since the full enactment of the ACA [19–21]. The uninsured rate among the U.S. nonelderly population has been reduced from 16.9 to 13 % between 2012 and 2015 [22, 23]. However, this evidence, albeit significant, falls short of our projections. A possible explanation for this discrepancy could be that some groups may continue to remain uninsured. Given the fact that most of the Southern states did not expand Medicaid coverage [24], uninsured individuals who live in these states and are eligible for Medicaid expansion (almost 50 % of the EME in this study) are more likely to remain uninsured with few options under the ACA. In addition, uninsured individuals with relatively low family incomes but not eligible for the Medicaid expansion (14.5 % of the uninsured) could be at risk for combined out-of-pocket expenses and premiums that are high relative to their incomes [25]. Lastly, individuals with high family incomes (7.9 % of the uninsured) could be more likely to choose to opt out due to the absence of federal subsidies. However, as penalties increase over time, this may be less likely [26].

In terms of health status, we found that the uninsured who were both RPIE and EME reported fewer health problems than those insured privately and publicly. Although these findings may reflect undiagnosed and untreated health conditions with limited access and unidentified barriers, respondents still reported a broad range of physical and mental conditions. Consistent with previous research on the effect of having coverage [6, 10, 27], expanded coverage with health insurance exchanges and the Medicaid expansion under the ACA would significantly increase access to preventive and primary care for the uninsured. Compared to the insured groups, the uninsured who were eligible for coverage reported less use of health care, including preventive health service, screenings (i.e., Pap smear, breast cancer screenings, and mammogram), and unmet health care needs, suggesting that having health insurance, regardless of coverage type, improves access to primary and preventive health care [11, 27].

However, the increased access to care under the ACA may also lead to more health disparities and lower quality of care between different types of coverage. We found that the publicly insured were more likely than the privately insured to experience difficulties obtaining health services when needed. Furthermore, despite the highest rates of health service usage, those publicly insured reported poorer physical and mental health conditions than not only those insured privately but also those uninsured, suggesting a wide variety of health service needs in current public health service providers. A study of the early Medicaid expansion in Oregon found that expanded Medicaid coverage had no effect on clinical outcomes and physical health status among newly insured while it increased health service use [28]. Another study showed that patients’ experiences with health service use were different according to types of insurance. Individuals in health maintenance organization (HMO) plans reported lower quality of care, lesser use of specialty care, and lower satisfaction with received care than their counterparts in non-HMOs [29]. Taken together with the results of this study, these findings suggest that the uninsured who were eligible for the Medicaid expansion (prospective publicly insured) could improve their access to healthcare services significantly, but perhaps not benefit as much with their health conditions.

### Limitations

Caution is warranted in generalizing the findings of this study to the general population. For instance, like many other studies, this study used self-reported measures. Therefore, the effects examined in the study may be limited to individuals’ perceptions of health status and health service utilization attributes. Although validity of self-reported measures have been proven useful [30], it is still possible that any systematic biases of recall and response affect precision of the estimates and the inclusion of questions in this study. Second, despite its national representativeness, the Medical Expenditure Panel Survey (MEPS) does not sample institutionalized populations. Thus, the results cannot be generalizable to individuals who are in institutions such as nursing homes or prisons. Third, this study presents a cross-sectional comparison, thus causality and validity of the results are not conclusive. Longitudinal studies are needed to confirm the relationship between types of coverage and health status over time. Lastly, it is likely that comparisons between the currently insured group and the group that would be likely to be insured (e.g., Public vs. EME [prospective publicly insured]) is limited and subject to interpretation without considering other possible confounding factors because they have the potential to be different population. Future studies will be needed to examine actual health outcomes for those newly insured under the ACA compared to their health outcomes prior to enactment.

## Conclusion

The ACA has increased coverage options and access to treatment and preventive health care services for the majority of the uninsured U.S. population. However, it may not play as significant a role in improving health among the uninsured, in particular, those who are eligible for the Medicaid expansion (EME). Ensuring access to preventive and mental health services is found to be important for addressing the needs of those who likely have coverage under the ACA. In particular, the publicly insured and the uninsured who were EME reported high rates of smoking, suggesting that efforts are needed to enhance the smoking cessation interventions for the publicly insured and those who are likely to get covered by the expansion of Medicaid.

Understanding the association between the types of insurance coverage with health status is critical to policy makers who must justify the ACA enactment. Policy makers should establish additional policies to ensure newly insured populations could receive quality care in both the private and public sectors. In addition, health providers must understand these findings so as to provide adequate care for those with different insurance types and those who are likely to be insured. Future research should explicitly consider how the types of coverage might influence health outcomes, and should track health disparities and inequalities between different types of health insurance coverage, including different deductible levels offered through the health insurance exchanges.

## Abbreviation

ACA: affordable Care Act
MEPS: medical Expenditure Panel Survey
SAQ: self-administered questionnaire
EME: the uninsured, but likely eligible for Medicaid
RPIE: the uninsured, but likely required to purchase health plans through the health insurance exchanges
CHAMPVA: civilian health and medical program of the department of veterans affairs
BMI: body mass index
AORs: adjusted odds ratios (AORs)
CI: confidence interval
HMO: health maintenance organization
